# Uninterrupted Use of Oral Anticoagulants for the Ablation of Atrial Flutter: A Single Center Cohort of 154 Patients

**DOI:** 10.5935/abc.20180001

**Published:** 2018-02

**Authors:** Tiago Luiz Luz Leiria, Alexandre Kreling Medeiros, Eduardo Dytz Almeida, Antonio Lessa Gaudie Ley, Catarine Benta Lopes dos Santos, Roberto Toffani Sant'Anna, Marcelo Lapa Kruse, Leonardo Martins Pires, Gustavo Glotz de Lima

**Affiliations:** Instituto de Cardiologia / Fundação Universitária de Cardiologia - IC/FUC, Porto Alegre, RS - Brazil

**Keywords:** Anticoagulants, Vitamin K, Catheter Ablation, Atrial Flutter, Thromboembolism

## Abstract

**Background:**

The uninterrupted use of oral anticoagulation (OAC) with vitamin K antagonists (VKAs) for electrophysiology procedures has been more and more recommended. The clinical practice in our service recommends the continuous use of these drugs for atrial flutter ablation. There is little evidence as to the uninterrupted use of non-vitamin K antagonist oral anticoagulants (NOACs) in this scenario.

**Objective:**

To compare the rates of complications related with the uninterrupted use of different types of oral anticoagulants in patients referred to atrial flutter (AFL) ablation.

**Methods:**

Historical, single-center cohort of ablation procedures by AFL conducted from November 2012 to April 2016. The primary outcome was the occurrence of hemorrhagic or embolic complication during the procedure. The secondary outcome was the occurrence of stroke or transient ischemic attack (TIA) in follow-up. The statistical significance level was 5%.

**Results:**

There were 288 ablations per AFL; 154 were carried out with the uninterrupted use of OAC (57.8% with VKA and 42.2% with NOAC). Mean age was 57 ± 13 years. The rate of hemorrhagic complication during the procedure was 3% in each group (p = NS). The rate of stroke/TIA was, respectively, of 56/1,000 people-year in the VKA group against zero/1,000 people-year in the NOAC group (p = 0.02).

**Conclusion:**

In our population there were no hemorrhagic complications regarding the procedure of OAC use uninterruptedly, including NOACs. There was higher occurrence of stroke/TIA in the follow-up of the group of patients undergoing VKAs; however, this difference may not only be a result of the type of OAC used.

## Introduction

The guidelines of the oral anticoagulant therapy^1^ recommend the suspension of these medications and the performance of heparin bridging, at the conduction of a wide range of invasive Cardiology procedures. Recently, the new classes of non-vitamin K antagonist oral anticoagulants (NOACs: rivaroxaban, apixaban, dabigatran and edoxaban) has proven to be effective to prevent the thromboembolic events in patients with atrial fibrillation (AF) and atrial flutter (AFL).^2^

The catheter ablation for AFL is a highly successful procedure in the reversion for the sinus rhythm.^3,4^ These cases require at least four weeks of anticoagulation before the procedure, as well as in electrical cardioversions, for the prevention of strokes or thromboembolic phenomena that can occur after the reversion of AFL to the sinus rhythm.^5^ Studies show that the use of NOACs seems to be safe in the prevention of these thromboembolic phenomena, for the reversion to the sinus rhythm.^6,7^

After the ablation, the use of anticoagulant is recommended for all patients for at least one month after the reversion to the sinus rhythm.^5^ The uninterrupted use of oral anticoagulant for AF procedures has proven to be safe^8,9^ ,and our institution adopts such a recommendation also for patients with AFL. Therefore, in this scenario, there are few studies carried out in Brazil.

The main objective of this study was to demonstrate the safety of the uninterrupted use of anticoagulation during flutter ablation, comparing the patients using NOACs with the vitamin K antagonists (VKAs). More specifically, we assessed the rate of hemorrhagic complications, as well as the occurrence of thromboembolic events throughout follow-up.

## Methods

Our study is a historical cohort that includes the procedures of ablation for AFL carried out in our Electrophysiology service (Instituto de Cardiologia Fundação Universitária de Cardiologia do Rio Grande do Sul). Of the 5,506 procedures conducted between November 2012 and April 2016, 288 (5.2%) corresponded to ablation for AFL. Data collection counted on the present description of the electrophysiological reports and with information obtained in an electronic and physical chart. The patients who discontinued the follow-up at the hospital outpatient clinic were selected for a telephone interview, and their consent was registered by the listener.

AFL was defined as a macro-reentrant atrial arrhythmia, electrocardiographically characterized by the presence of F-waves with constant morphology, and atrial frequency higher than 250 bpm. AFL typical was considered when the electrocardiogram (ECG) showed negative F-waves in derivations DII, DIII and VF, and positive in V1.^3^

The parameters of the left ventricular ejection fraction (LVEF) and left atrial (LA) diameter were collected by the most recent echocardiogram found in the records, that had been conducted before ablation, which includes both transesophageal (TEE) and transthoracic (TTE) examinations. The ejection fraction was calculated using the methods of Teichholz or Simpson, according to the presence of segmental dysfunction. The atrial diameters were assessed using the M mode.

The charts were revised aiming at recording the clinical information that was necessary for points in the score of CHA_2_DS_2_VASc (congestive heart failure, hypertension, age, diabetes, stroke, vascular disease, and female gender): sex, age, diagnosis of systemic arterial hypertension (SAH), diabetes mellitus (DM), congestive heart failure (CHF) or LVEF < 50%, peripheral vascular disease, myocardial infarction or aortic atherosclerosis and history of stroke or TIA. The referred diagnoses were defined according to previous publications.^10^ Data of anticoagulation were registered before the ablation.

The patients who were receiving the same medication in the four weeks prior to the procedure were considered as undergoing uninterrupted use: VKAs (warfarin and phenprocoumon), with international normalized ratio (INR) between 2 and 3.5, and NOACs (dabigatran, rivaroxaban and apixaban). All patients received the dosage of NOAC on the day before the procedure, in the morning or in the afternoon, at the assistant physician’s choice and according to the posology of the NOAC used (once or twice a day). None of the cases was performed with an interval higher than 24 hours after the administration of the daily use NOACs, or 12 hours after the ones with double dosage. The dose on the day of the ablation was instituted four hours after the removal of the introductory sheaths. The patients on VKA received the dose of the medication four hours after the removal of the introducers.

The patients were followed-up at the outpatient clinic, and the first appointment was conducted from one to three months after ablation, through a clinical visit and 12-lead ECG. During the follow-up of these patients, we also included the data referring to emergency care or hospitalizations that took place in our institution.

The patients who discontinued the follow-up at the outpatient clinic were selected for a telephone interview to clarify the following:

- If they continued to use the anticoagulant;
- If they presented an episode of stroke or TIA;
- If they had any late complications related with the procedure.

The Research Ethics Committee of our hospital approved the study protocol and we obtained a consent from all listeners for the performance of the interview. The study’s protocol n. is UP 5252/16.

### Outcomes

We defined the following as main outcomes: the occurrence of hemorrhagic complication during the procedure; some examples are cardiac tamponade, bleeding that requires transfusion, bleeding with reduction of ten percentage points in the hematocrit, local vascular complication requiring intervention (major hemorrhagic events), and clinically uncomplicated hematomas (minor hemorrhagic event); adverse heart events were considered as a compound of all mortality causes, stroke, TIA, during follow-up.

One specialist of each field validated each outcome.

### Exclusion criteria

All patients with AFL submitted to a second procedure were excluded, as well as those with history of previous ablation at another service, those with left AFL and those who did not undergo the uninterrupted use of OAC in the peri-procedural period. The patients using low-molecular-weight heparin (full anticoagulant dose or unfractionated heparin in continuous intravenous infusion, even if anticoagulated) were not included in this study.

### Statistical analysis

The data were stored and analyzed with the Statistical Package for the Social Sciences (SPSS), version 22.0 (SPSS Inc., Chicago, IL, USA). The continuous variables were expressed as mean ± standard-deviation, and compared by the Student’s *t* test for independent samples. The categorical variables were expressed in percentage and compared using the χ^2^ test. The variables were considered normal according to the observation of the central tendency measurements, kurtosis and asymmetry in the frequency histograms. The incidence density was calculated using the people-time interval for the occurrence of thromboembolic phenomena in the post-ablation follow-up. This measure was carried out combining the number of people and the contribution of time during the study, and it was used as a denominator in the incidence rates. It was defined as the sum of individual units of time to which the people in the population studied were exposed, or at risk for the outcome of interest. The statistical significance level adopted was 5%.

## Results

**Figure 1:**
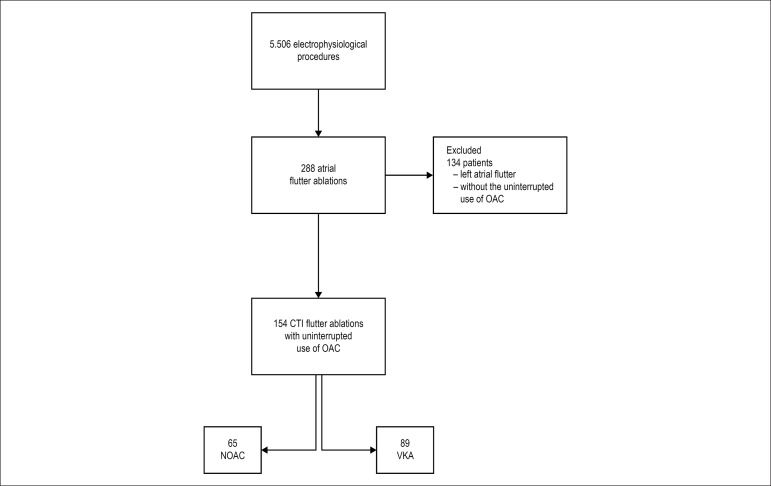
Study flowchart. CTI: cavotricuspid isthmus dependent flutter; OAC: oral anticoagulation; NOAC: non-vitamin K antagonist oral anticoagulants; VKA: vitamin K anticoagulant antagonists.

In the study period, there were 288 ablations per AFL. Of these, 154 were conducted with the uninterrupted use of oral anticoagulants, and these cases were included in the study. Figure 1 demonstrates the organization chart of inclusion of cases in the study. The mean age was 57.3 ± 13.1, and most were male (70%). The mean CHA_2_DS_2_-VASc was 2.1 ± 1.5 points, and 63% had a score higher than or equal to 2. Of the ablations, 98% were carried out with an 8 mm catheter - only 2% were conducted with an irrigated catheter.

The VKAs were used uninterruptedly in 57.8% of the cases, and NOACs, in 42.2% of the participants. The mean INR was 2.54 ± 0.54 in the VKA group on the day of the ablation. The patients using NOAC were the majority at a sinus rhythm on the day of the ablation. These patients had smaller left atriums. Besides, they also used more antiarrhythmic drugs, less beta-blockers and statins, with lower prevalence of previous heart surgery when compared to patients using VKA. Table 1 shows the clinical characteristics of the patients stratified by type of anticoagulant used. Table 2 exemplifies the frequency of use of different types of NOACs and VKAs used in the study.

**Table 1:** Difference between the populations that received vitamin-K antagonists and the ones who received non-vitamin K antagonists uninterruptedly for atrial flutter ablation

Factor	NOAC (n = 65)	VKA (n = 89)	p value
Previous history of AF	23 (35.4%)	28 (31.5%)	0.77
Age (years)	58.1 ± 11.7	56.8 ± 14.1	0.55
Gender (male)	45 (69.2%)	63 (70.8%)	0.97
Sinus basal rhythm	33 (50.8%)	28 (31.4%)	0.02
LVEF (%)	59.6 ± 12.3	58.0 ± 16.6	0.57
LA (mm)	44.3 ± 6.2	47.7 ± 7.7	0.01
CHA_2_DS_2_VASc ≥ 2	64.6%	61.8%	0.852
- SAH	59.4%	73.0%	0.07
- DM	20.6%	20.2%	0.95
- Stroke	9.5%	3.4%	0.113
Beta-blockers	55.4%	79.8%	0.002
Calcium channel blockers	10.8%	13.5%	0.79
ACEi/ARB	44.6%	55.1%	0.26
Diuretics	29.2%	41.6%	0.16
Digoxin	12.9%	14.9%	0.90
Statins	27.7%	44.9%	0.04
ASA	15.4%	28.1%	0.09
Antiarrhythmic drugs	55.4%	33.7%	0.01
Previous heart surgery	7.7%	38.6%	< 0.001
- Valvar	0.0%	22.7%	0.0001
Ischemic cardiopathy	10.8%	19.3%	0.22
Congenit cardiopathy	9.2%	9.1%	0.79
Myocardiopathy	10.8%	19.3%	0.22
COPD	3.0%	7.9%	0.36

NOAC: non-vitamin K antagonist oral anticoagulants; VKA: vitamin K anticoagulant antagonists; AF: atrial fibrilation; LVEF: left ventricular ejection fraction; LA: left atrium; CHA2DS2VASc: risk for stroke (congestive heart failure, hypertension, age, diabetes, stroke, vascular disease, and female gender); SAH: systemic arterial hypertension; DM: diabete mellitus; ACEi/ARB: angiotensin-converting enzyme inhibitors / angiotensin receptor blocker; ASA: acetylsalicylic acid; COPD: Chronic obstructive pulmonary disease. The p value expresses the difference of the Student's t test for the continuous variables and the χ^2^ in the categorical variables. The statistical significance level adopted was 5%.

**Table 2:** Type of non-vitamin K antagonist oral anticoagulants and vitamin K anticoagulant antagonists used uninterruptedly for the atrial flutter ablation

NOAC (n = 65)%	VKA (n = 89)%
Rivaroxaban (41) 63.0%	Warfarin (77) 86.5%
Dabigatran (14) 21.6%	Phenprocoumon (12) 13.5%
Apixaban (10) 15.4 %	

NOAC: non-vitamin K antagonist oral anticoagulants; VKA: vitamin K antagonist.

The rates of hemorrhagic complication related with the procedure was 3% in each group (p = 0.97). There were no cases of cardiac tamponade or major hemorrhagic complication in the patients of the study. The main complications related with the procedure were inguinal hematomas. The rate of stroke / TIA was 57/1,000 people-year in the VKA group against zero/1,000 people-year in the NOAC group (p = 0.02).

## Discussion

Our study shows the safety of the use of oral anticoagulants (VKAs or NOACs) in the periprocedural period of the radiofrequency ablation of typical AFL. The use of periprocedural anticoagulation is based on the frequent finding of atrial thrombi or of spontaneous echo contrast in the transesophageal echocardiogram.^11^ The studies about the oral anticoagulation in these patients, however, are scarce, and there are no clear recommendations in the guidelines about the handling of periprocedural anticoagulation for the ablation of AFL.^8,12-14^

A retrospective study with 254 patients, comparing periprocedural warfarin and dabigatran of ablation of AFL and AF, demonstrated similar results to that of our cohort, with low rates of thromboembolic and hemorrhagic complications. However, the authors do not show the number of patients with AFL included in the study.^12^

A second retrospective study with 60 patients who used dabigatran or rivaroxaban in the periprocedural period of AFL ablation demonstrated low incidence of hemorrhagic complications, with 4 minor bleedings (3 of the 23 patients using dabigatran 150 mg b.i.d, and 1 of the patients using rivaroxaban 20 mg), and no major bleeding.

A second retrospective study with 60 patients who used dabigatran or rivaroxaban in the periprocedural period of AFL ablation demonstrated low incidence of hemorrhagic complications, with 4 minor bleedings (3 of the 23 patients using dabigatran 150 mg b.i.d. and 1 of the 11 patients using rivaroxaban 20 mg), and no major bleeding. A patient using dabigatran 110 mg b.i.d. presented with ischemic stroke 27h after the procedure, in the uninterrupted use of anticoagulant, with preprocedural transesophageal echocardiogram that did not show atrial thrombi. This study, however, collected data only until the hospital discharge of the patients; therefore, the security data of the use of these medications may be underestimated.^15^

A third retrospective study of NOACs in this scenario compared patients using apixaban (n = 105), paired with others that used phenprocoumon (n = 210) until hospital discharge.^13^ Only the patients submitted to ablation of left atrial arrhythmia were included, unlike our cohort, which only included cases of typical flutter. All patients were using oral anticoagulation for at least four weeks, and the use of anticoagulation was uninterrupted, with use of endovenous heparin during the procedure. There were no thromboembolic events; minor bleedings occurred in 10.5% of the patients using apixaban, and in 12.3% of those using phenprocoumon (p = 0.61). Our cohort demonstrated fewer hemorrhagic complications, however, no procedure carried out approached the left atrium.

Proving the variability in the handling of periprocedural anticoagulation of AFL ablation, a study conducted in Europe and in Canada showed that 6% of the centers do not use routine anticoagulation in typical AFL ablation, and that only 31% of the centers performed preprocedural anticoagulation for a minimum period of 4 weeks.^16^ Regarding the use of NOACs, only 35% of the centers perform the procedure with the uninterrupted use of medication, and those who suspended the medication demonstrate great variation in the period of the suspension.

The increasing use of NOACs since 2010, as demonstrated by the study GARFIELD-AF,^17^ points to the need of data collection regarding the use of these classes of drugs in the most varied scenarios. The scenario of AFL ablation, however, requires prospective studies that are able to unify the conducts of the electrophysiology centers. Our study points to the security of these drugs and paves the way for the clinical trials to be conducted.

One point to be emphasized in our study was the almost exclusive use of the 8 mm ablation catheter, which may not reflect the reality of other services. There is a sensation that, in the region of the cavotricuspid isthmus, whose thickness ranges between 0.5 and 5 mm,^18,19^ the application of high energy (70 W) may lead to an increasing risk of perforation. However, the studies that assessed the use of 8 mm catheters, in comparison to irrigated ones, in the ablation of isthmus-dependent AFL, demonstrated there were no significant differences in the occurrence of vaporization lesions (“pop”) or cardiac perforation.^20-22^ The occurrence of carbonization at the end of the catheter, however, seems to be higher than in the irrigated catheter,^20^ but this fact was not measured in our study.

### Study limitations

As limitations of our study, we mentioned that part of data collection was conducted retrospectively, through the analysis of medical records, which could lead to bias in the confirmation of the outcomes. However, our center presents a routine of peri and post-procedural care, which contemplates the collected variables, which mitigates the potential bias. Also, the number of patients analyzed may not have been sufficient to detect a statistically significant difference between the groups regarding the lower incidence outcomes. Another important aspect is that, even though the incidence density for ischemic events was higher in our study in the VKA group, this does not mean that one strategy is superior to another in the post-ablation period. As demonstrated, the patients using VKA have different characteristics than those using NOAC. The comparison between two distinct groups of patients is a significant limitation of this study. Besides the bias caused by the retrospective design, the VKA group presents almost 23% of the etiology patients (against none in the NOAC group). The valvar patients clearly presented with higher thromboembolic risk. Also, since this is an observational design, strategies for the strict control of therapeutic-target achieving (TTA) time were not conducted, and studies carried out in our service demonstrated mean TTA of about 50% in our population.^23^

## Conclusion

This historical cohort points to the safety in the conduction of radiofrequency ablation of typical AFL procedures with the uninterrupted use of oral anticoagulants, regardless of the class of this group of medication.
